# Thoughts at the beginning of the term

**Published:** 2012-06-18

**Authors:** I Sinescu

**Affiliations:** Carol Davila University of Medicine and PharmacyRomania

The mission I was assigned, that of leading the biggest and the oldest school of medicine in Romania, is an honorable task, but, in the same time, it is very difficult. 

First, because we are going through a complex period, in which a change is requested and, most importantly is needed, but the reforms are, very often, misunderstood or inapplicable, and the future depends on their approach and implementing. The essential changes must be identified starting from the realistic estimation of their utility and complexity, the change in the medical world requiring the identification of new methods of innovation in the healthcare services provision. 

Secondly, because “Carol Davila” University of Medicine and Pharmacy is an inexhaustible reservoir of reference medical personalities in the Romanian Medicine, it must keep on “blooming” at the same standards of professionalism and performance, an obligation that is extremely serious and difficult to achieve. 

Thirdly, as a result of the fact that I am the successor of a rector, who, in the two terms, the maximum permitted by the Romanian laws, has not only been respected but also loved by the entire academic community. 

Just as I promised, the fundamental objective of my preoccupations will be the acceleration of the efforts of transforming “Carol Davila” University of Medicine and Pharmacy in an elite university, ranked in the top 500 universities of the world. I intend to do this by approaching a responsible, open and flexible attitude of the leaders of the universities at all the levels: discipline, department, university council or university senate, assuring a balance between tradition and innovation, academic excellence and clinical efficiency, the coherence of the curricula and the students’ freedom of choice, in order to form some competitive doctors, true professionals, capable of integrating in this perpetual competition in the fight for health. 

All these cannot be accomplished without a quality education, centered on the student, without the continuous improvement of the learning process by modern teaching methods, fair partnership relationships with the students, encouragement of the initiative, creativity, dialogue and the effective and active implication of the students in the instructive educative theoretical and practical process, without the qualitative growth of the scientific research activities. 

Therefore, just as I stated before, as a representative of our university community, I have the obligation of maintaining a communication channel accessible to each member belonging to it: student, researcher or professor, an equal access according to the laws specific to promotion contests and, where it is the case, protection against the abuse of authority. I am confident that I can transform the principles which have led my professional progress and have represented the base of my career, *a well administration-transparency-collegiality*, in the directory lines of a managerial activity, which is worthy of our university. 

The challenge consists in realizing such a thing in a prestigious academic institution, so that the preparation of the new generations is made with firm steps towards the modernization and the efficiency of the medical teaching system, by continuing the permanent raise of its quality on the background of developing the working cult, the solidarity among people who share the same occupation and the medical honor.

